# Supplementary material for: Identification of catalytic sites in cobalt-nitrogen-carbon materials for the oxygen reduction reaction
Source: Nat Commun. 2017 Oct 16;8:957. doi: 10.1038/s41467-017-01100-7 (PMC5715157; doi:10.1038/s41467-017-01100-7)
Supplement: Supplementary file 1 — Supplementary Information [file 41467_2017_1100_MOESM1_ESM.pdf]

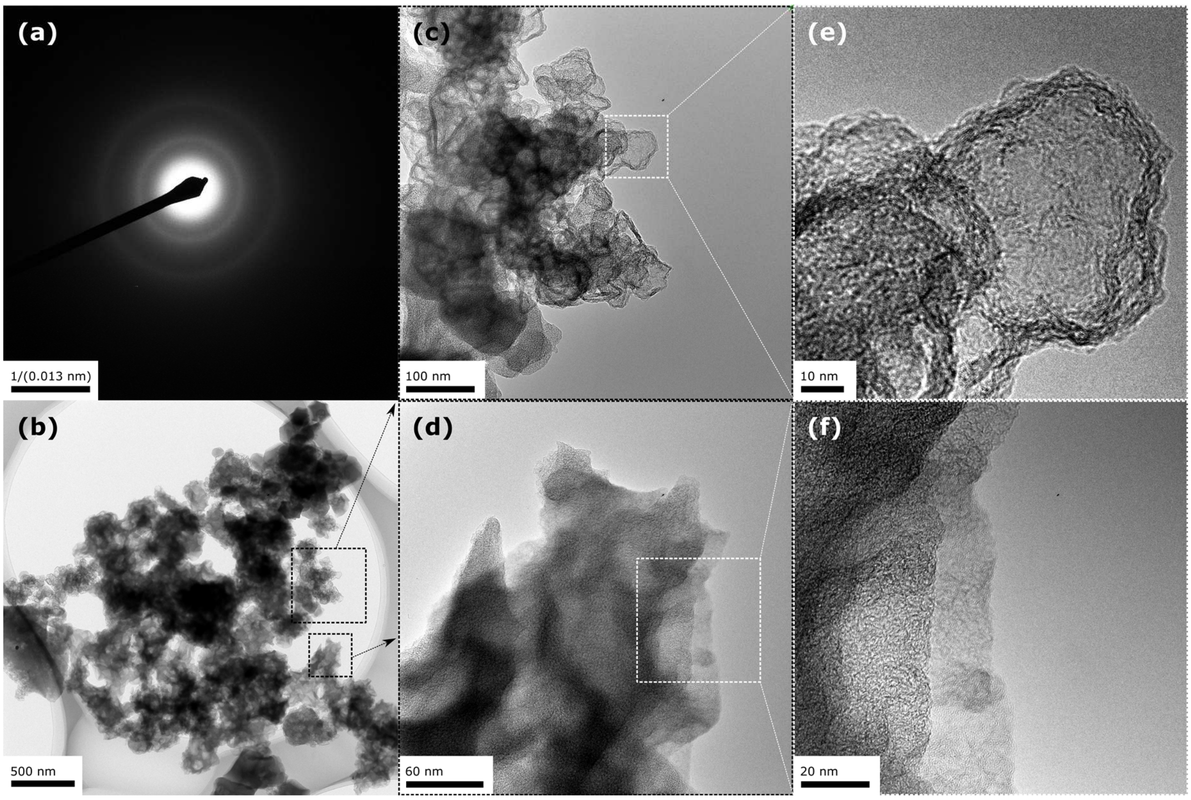

Supplementary Figure 1. TEM analysis of $Co_{0.5}$ showing (a) a SAED pattern, and (b-f) bright-field images of the microstructure. Only two broad rings were observed in the SAED pattern, as expected for amorphous carbon. Metallic nanoparticles would appear as sharp rings or discrete spots in the SAED, and therefore this provides strong evidence that cobalt particles are absent in this material. Two different morphologies were observed in the microstructure of the catalyst. Images in (c) and (e) show graphitic domains in the walls of hollow structures. In contrast, the microstructure in images (d) and (f) is more sheet-like, and several carbon layers can be seen at the edges of the structure.

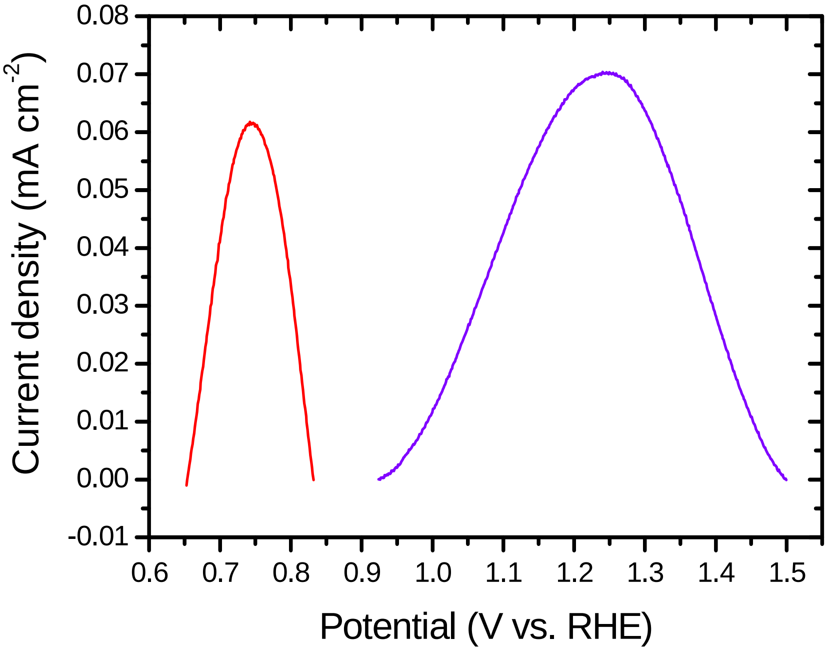

Supplementary Figure 2. Position of redox peaks identified by square-wave voltammetry (red: $Fe_{0.5}$, purple: $Co_{0.5}$). Measured in 0.1 M $HClO_4$.

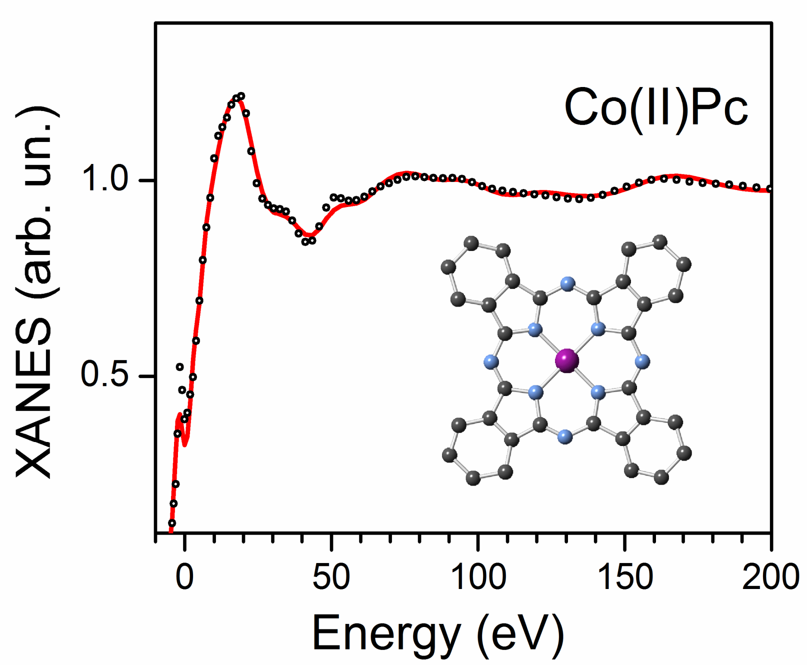

Supplementary Figure 3. Comparison between the Co K-edge XANES experimental spectrum of Co(II) phthalocyanine (black hollow circles) and the theoretical spectrum calculated from its known structure (solid red line). The purple sphere represents a cobalt atom, while blue and grey spheres identify nitrogen and carbon atoms, respectively.

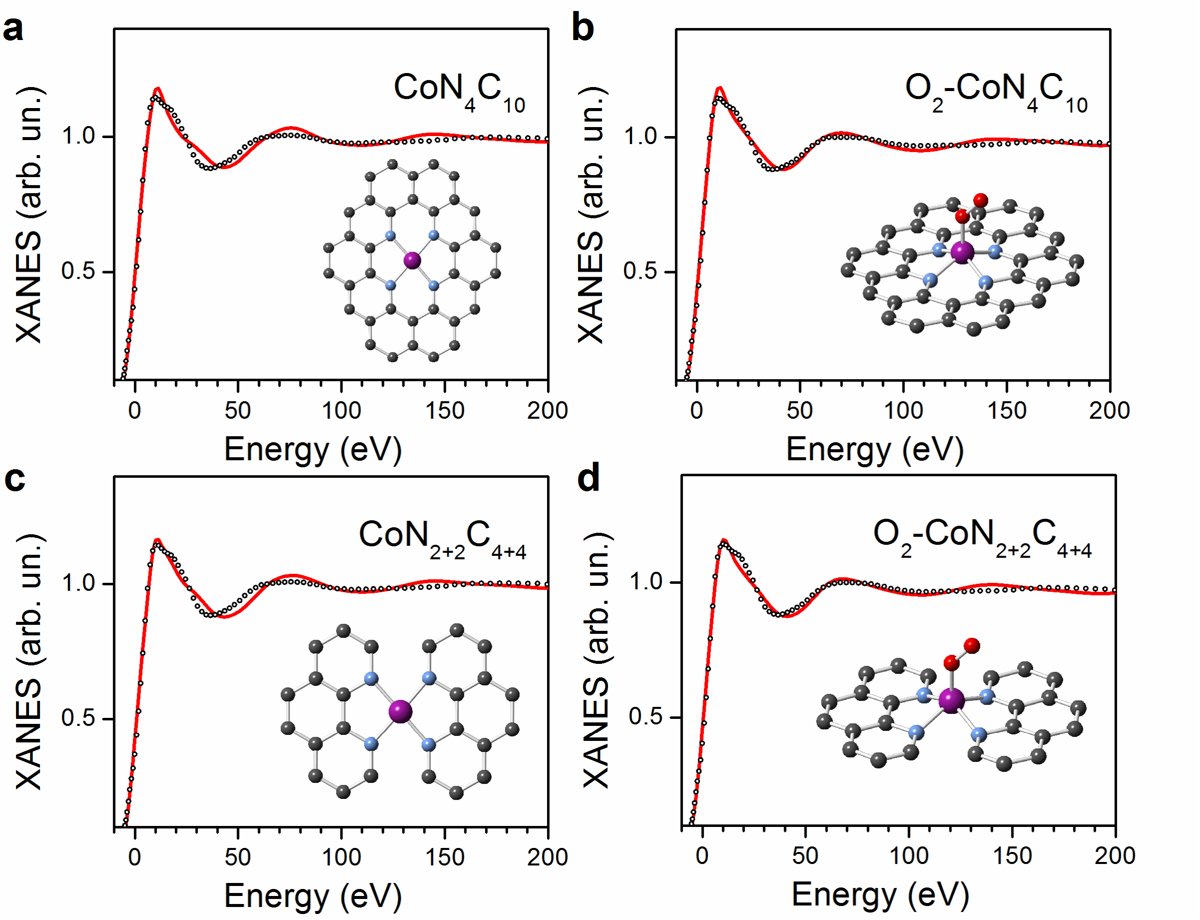

Supplementary Figure 4. Comparison between the K-edge XANES experimental spectrum of $Co_{0.5}$ (black hollow circles) and the theoretical spectrum calculated with the depicted structures (solid red lines). The purple sphere represents a cobalt atom, while blue and grey spheres identify nitrogen and carbon atoms, respectively

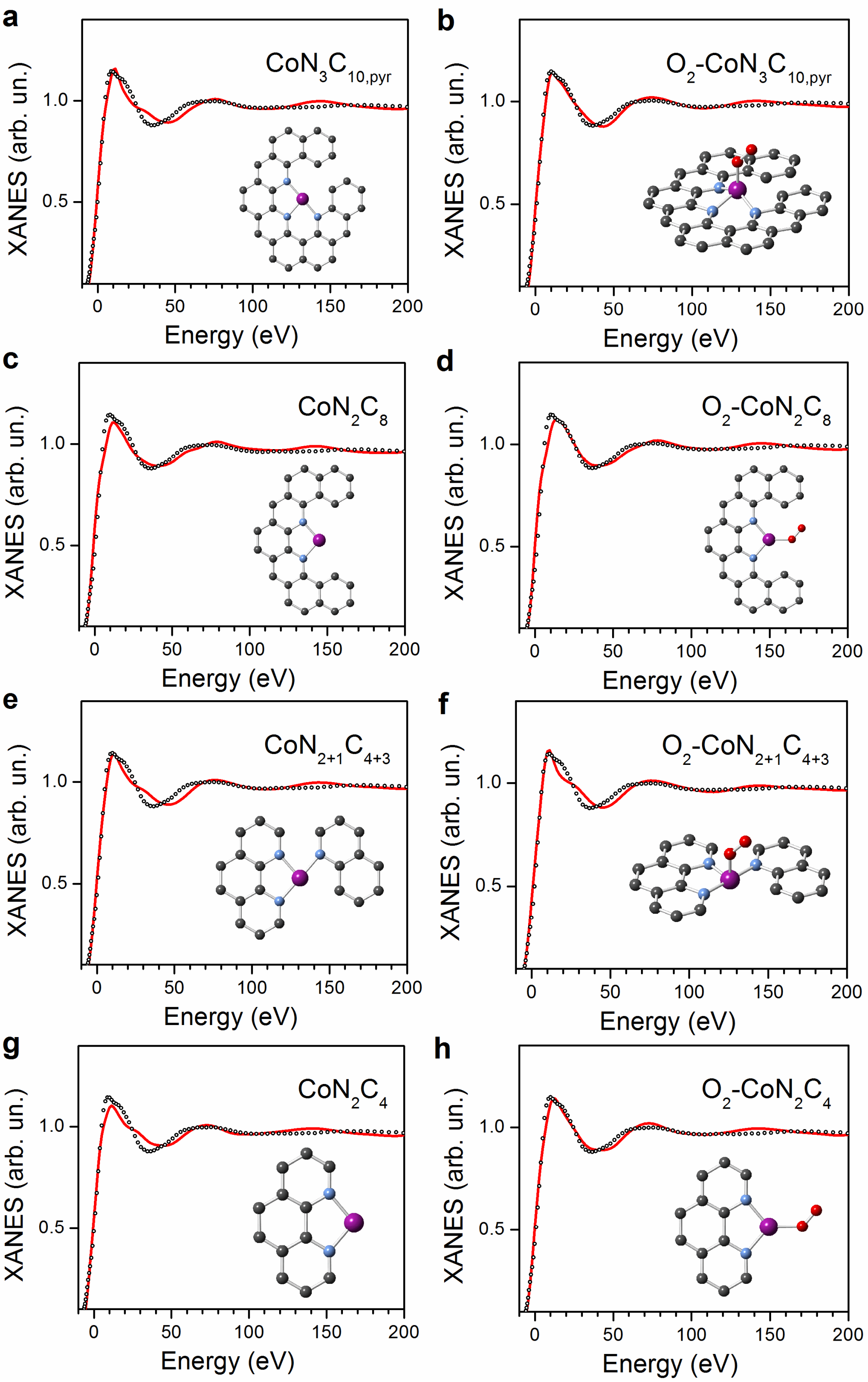

Supplementary Figure 5. Comparison between the K-edge XANES experimental spectrum of $Co_{0.5}$ (black hollow circles) and the theoretical spectrum calculated with the depicted structures (solid red lines). The purple sphere represents a cobalt atom, while blue and grey spheres identify nitrogen and carbon atoms, respectively.

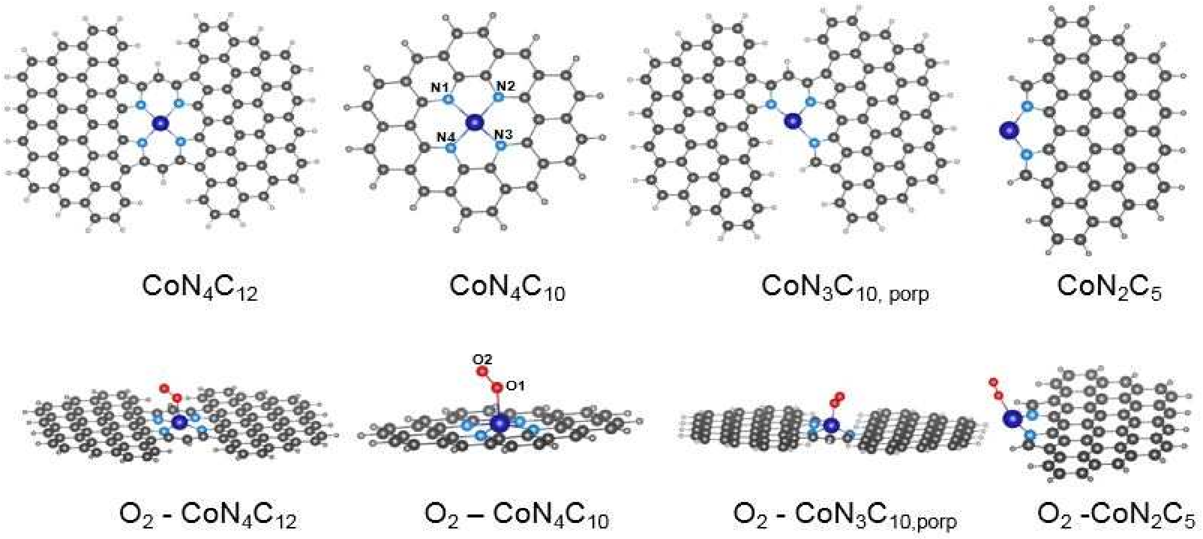

Supplementary Figure 6. DFT-D optimised cluster models

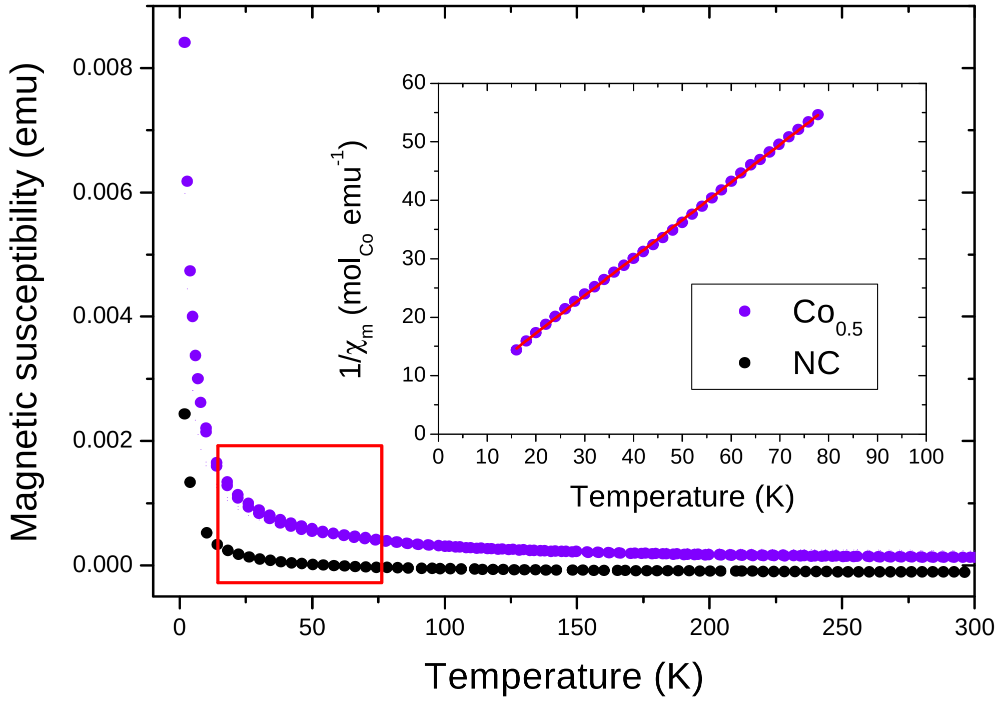

Supplementary Figure 7. Magnetic susceptibility of $Co_{0.5}$ and N-C as a function of temperature**.** Inset: The linear fitting of $1/\chi_m$ as a function of temperature was performed in the region 15-77 K where the absolute signal of N-C is < 10 % that of $Co_{0.5}$ (the fit region is indicated by the red square).

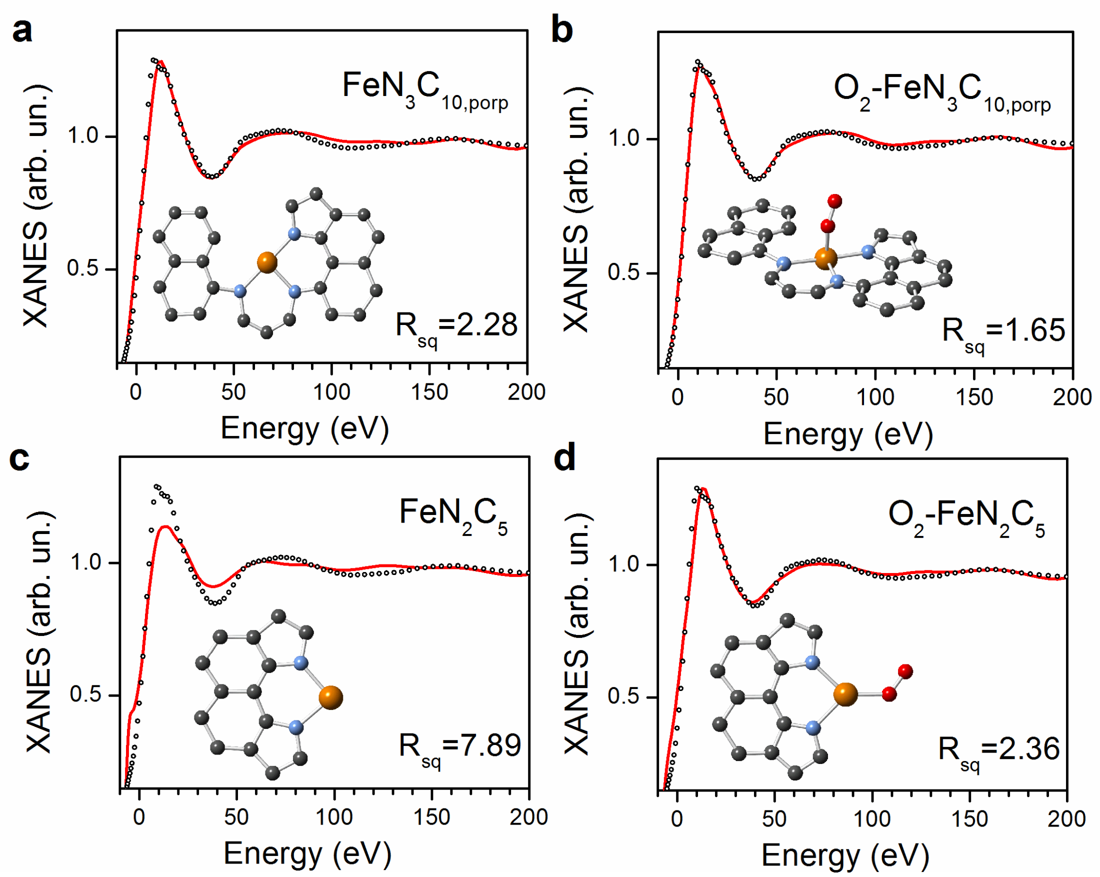

Supplementary Figure 8. Comparison between the K-edge XANES experimental spectrum of $Fe_{0.5}$ (black hollow circles) and the theoretical spectrum calculated with the depicted structures (solid red lines). The brown sphere represents an iron atom, while blue and grey spheres identify nitrogen and carbon atoms, respectively.

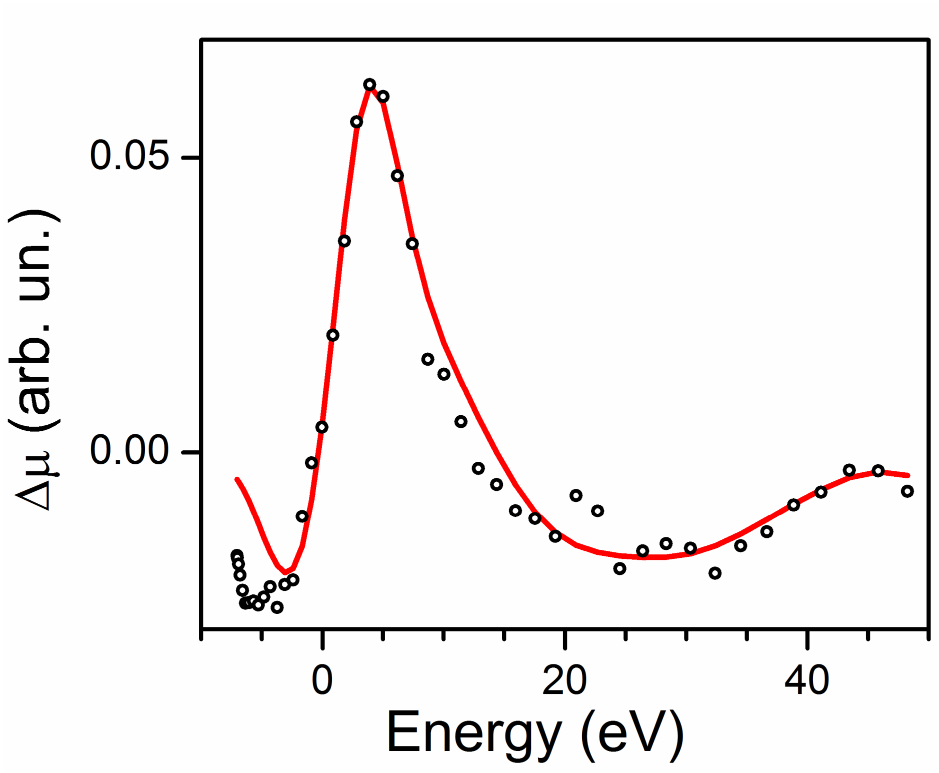

Supplementary Figure 9. XANES theoretical fit (solid red lines) of the experimental Δμ spectrum of $Co_{0.5}$ (black hollow circles) obtained by subtracting the XANES spectra measured in N_2- and O_2-saturated acid electrolyte at 0.8 V.

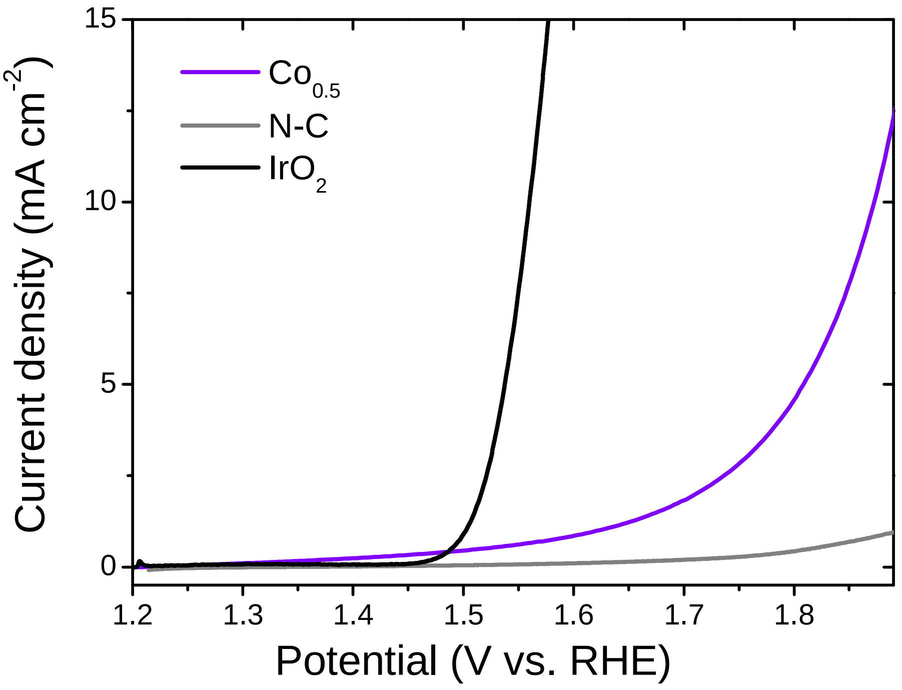

Supplementary Figure 10. Oxygen evolution reaction on $Co_{0.5}$, N-C and IrO_2 in acid medium. The electrolyte was an O_2-saturated 0.5 M H_2SO_4 ($Co_{0.5}$ and N-C) or 0.1 M H_2SO_4 (IrO_2) aqueous solution. The total catalyst loading (all elements) for $Co_{0.5}$ and N-C was 0.8 mg cm^{-2} and for unsupported IrO_2 it was 0.6 mg cm^{-2}. All measurements were performed at 1600 rpm with a scan rate of 10 mV s^{-1}. In addition, $Co_{0.5}$ and N-C have similar BET surface areas and hence capacitive currents. Unsupported IrO_2 was prepared following a hydrolysis method. Briefly, H_2IrCl_6 was added to 0.5 M NaOH and heated to 80°C. The solution pH was then decreased to 8.0 using 1 M HNO_3 and kept at 80°C for 30 min. The obtained powder was washed and centrifuged with water till neutral pH was obtained. The remained slurry was dried at 80°C, then crushed with a pestle and mortar, and calcined in air at 500°C for 30 minutes.

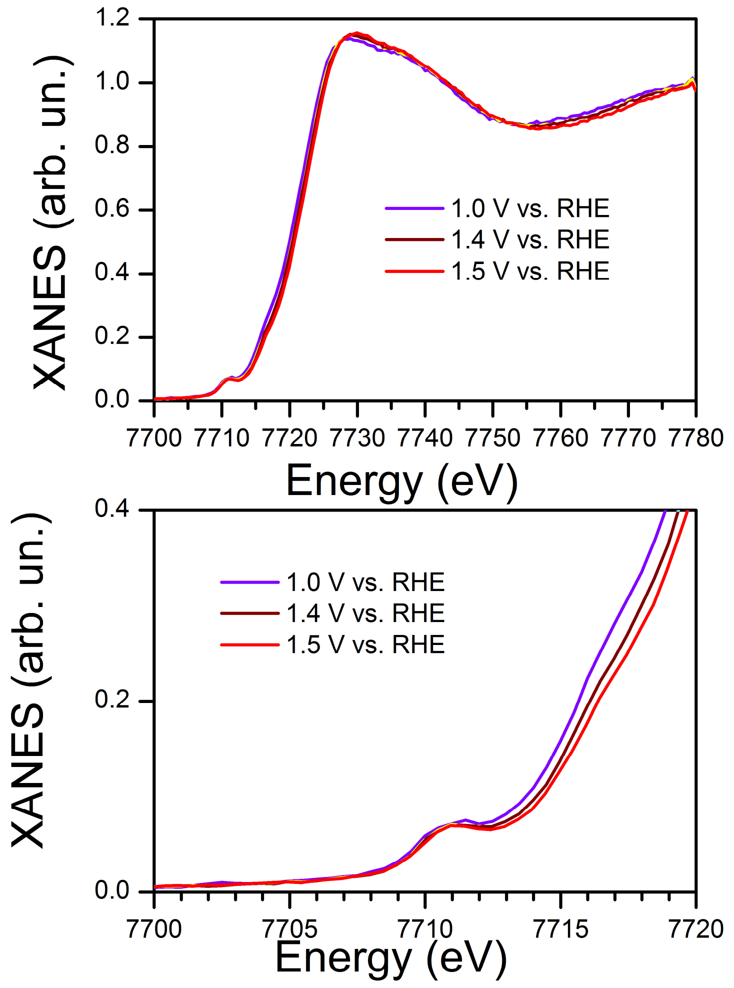

Supplementary Figure 11. *Operando* XANES spectra in 0.5 M H_2SO_4 for $Co_{0.5}$ at OER potential. Top: full XANES spectra, bottom: zoom showing the near edge region. The spectra were measured as a function of the electrochemical potential in N_2-saturated electrolyte.

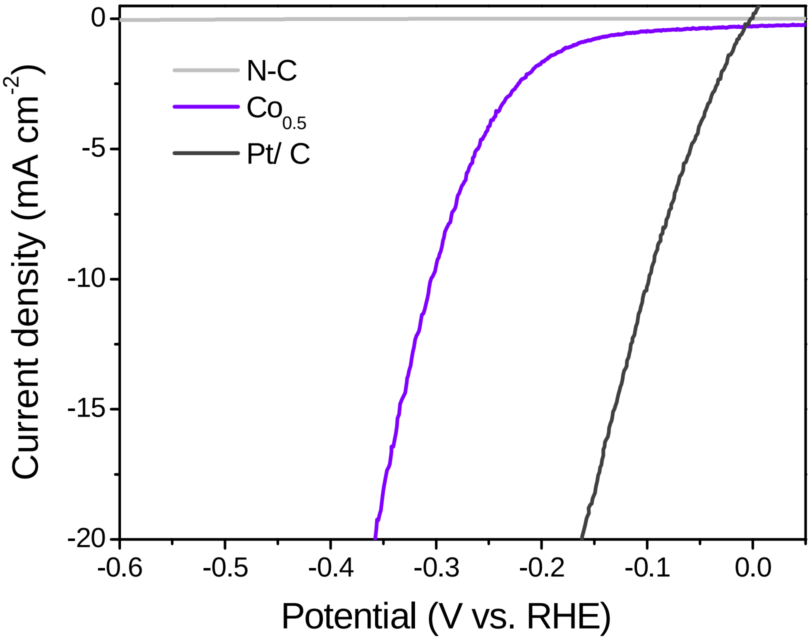

Supplementary Figure 12. Hydrogen evolution reaction on $Co_{0.5}$, N-C and Pt/C in acid medium.

The electrolyte was an H_2-saturated 0.5 M H_2SO_4 aqueous solution. The total catalyst loading (all elements) for $Co_{0.5}$, N-C and Pt/C was 0.8 mg cm^{-2} (5 wt % Pt on C, resulting in 40 μg_{Pt} cm^{-2}). All measurements were performed at 1600 rpm with a scan rate of 10 mV s^{-1}.

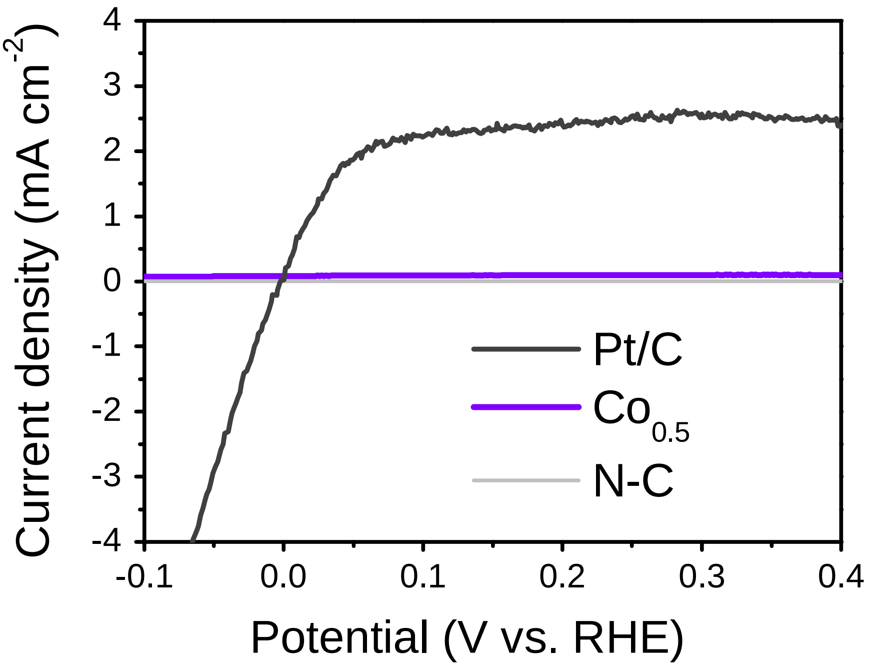

Supplementary Figure 13. HOR activity for $Co_{0.5}$, N-C and Pt/C in H_2-saturated 0.1 M H_2SO_4 solution.

The total catalyst loading (all elements) for $Co_{0.5}$, N-C and Pt/C was 0.8 mg cm^{-2} (5 wt % Pt on C, resulting in 40 μg_{Pt} cm^{-2}). All measurements were performed at 1600 rpm with a scan rate of 10 mV s^{-1}.

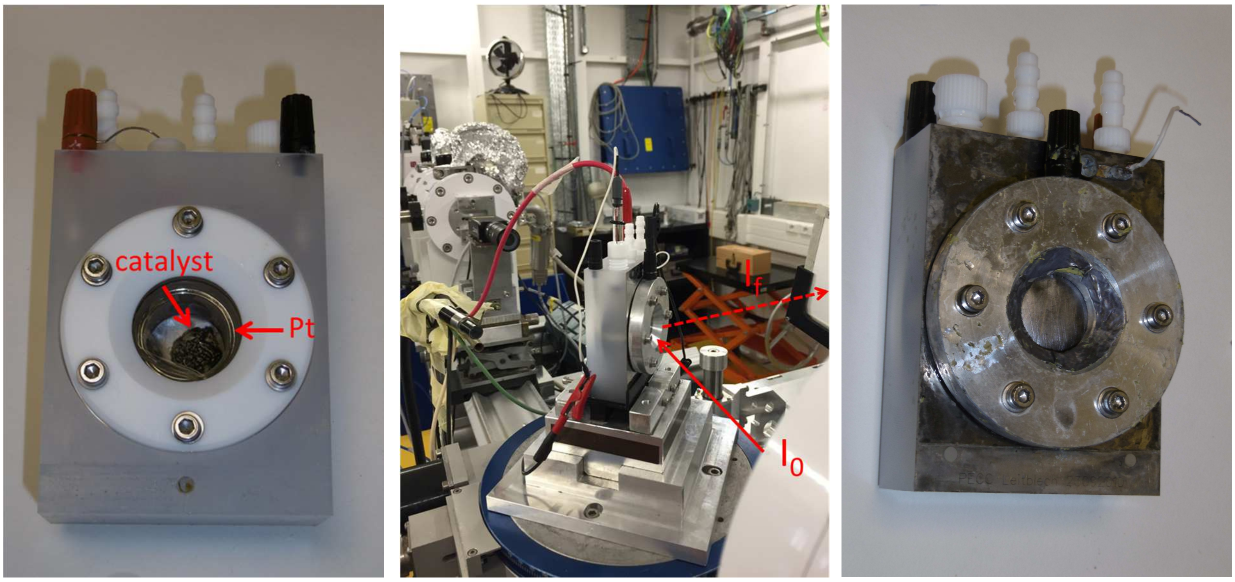

Supplementary Figure 14. *Operando* XANES electrochemical cell. Left: back of the cell showing the Pt counter electrode coil and the Me-N-C catalyst ink deposited on the side of the graphite foil facing the electrolyte. Middle: experimental setup used for recording fluorescence XANES spectra. Right: front of the cell showing the graphite foil working electrode facing the beam.

Supplementary Table 1. Best-fit parameters obtained from the analysis of the EXAFS spectrum of $Co_{0.5}$

	CN	R(Å)	σ^2 (Å^2)	β
4-fold coordination				
Co-N	3.8(6)	1.96(2)	0.008(2)	0.8(2)
5-fold coordination				
Co-N	4.0(6)	1.95(2)	0.007(2)	0.7(2)
Co-O	0.7(1)	2.16(3)	0.020(3)	0.7(2)

CN is the coordination number, R is the interatomic distance, σ^2 is the Debye-Waller factor and β the asymmetry parameter. Errors are given in brackets.

Supplementary Table 2: Best-fit structural parameters obtained from the analysis of the XANES spectrum of $Co_{0.5}$ performed on the structures depicted in Supplementary Figure 4 and Supplementary Figure 5.

Moiety	Co-N / Å	Co-O / Å	bending / °	R_{sq}
**CoN_4C_{10}**				
4-fold	1.96 (3)	--	--	2.97
5-fold	2.00 (3)	1.82 (4)	63 (4)	2.65
**$CoN_{2+2}C_{4+4}$**				
4-fold	1.95 (2)	--	--	2.81
5-fold	2.01 (3)	1.78 (5)	43 (5)	2.70
**$CoN_3C_{10,pyr}$**				
3-fold	1.98 (3)	--	--	3.13
4-fold	2.00(3)	1.85(4)	42(7)	2.67
**CoN_2C_8**				
2-fold	1.96 (3)	--	--	3.08
3-fold	1.98(2)	1.89(4)	50(5)	2.93
**$CoN_{2+1}C_{4+3}$**				
3-fold	1.96 (3)	--	--	3.10
4-fold	1.97(3)	1.80(5)	43(4)	2.90
**CoN_2C_4**				
2-fold	2.00 (3)	--	--	3.25
3-fold	2.01(3)	1.90(4)	45(5)	2.78

R_{sq} is the residual function. Errors are given in parentheses.

Supplementary Table 3. Selected DFT-D optimised bond distances and angles for the ground-state structures.

	CoN_4C_{12}	CoN_4C_{10}	CoN_3C_{10}porp	CoN_2C_5	O_2-CoN_4C_{12}	O_2-CoN_4C_{10}	O_2-CoN_3C_{10}porp	O_2-CoN_2C_5
Co-N1 / Å	2.020	1.910	-	-	2.020	1.931	-	-
Co-N2 / Å	2.038	1.908	2.206	1.964	2.051	1.912	2.373	2.009
Co-N3 / Å	2.052	1.908	1.888	1.976	2.100	1.918	1.887	2.048
Co-N4 / Å	2.019	1.910	1.930	-	2.069	1.921	1.935	-
N1-Co-N2 / °	86.0	86.9	-	-	84.6	86.2	-	-
N3-Co-N4 / °	84.2	87.0	90.5	-	84.0	86.1	91.9	-
N1- Co -N4 / °	94.9	92.8	-	-	94.0	91.4	-	-
N2- Co -N3 / °	94.9	92.8	98.1	112.8	95.3	92.4	96.1	106.1
Co -O / Å					2.024	1.97	1.771	1.782
O-O / Å					1.292	1.31	1.329	1.329

For the considered cluster models and atom numbering, see Supplementary Figure 6. In O_2-CoN_3C_{10}porp and O_2-CoN_2C_5 moieties, Co is displaced out of plane by 0.4 and 0.5 Å, respectively

.

Supplementary Table 4. Relative energy (ΔE), spin of the moiety, Mulliken spin density of cobalt and oxygen atoms in O_2 (O1 is bound to cobalt), Co(II) binding energy (BE) and O_2 adsorption energy in end-on mode (E_{ads}).

Moiety	Spin of moiety	Spin density of cobalt	Spin density of O1 / O2	ΔE / eV	BE / eV	E_{ads} / eV
CoN_4C_{12}	**1/2**	**0.88**		**0.00**	**-7.48**	
	3/2	1.12		0.31	-7.40	
	5/2	2.50		0.83	-7.27	
CoN_4C_{10}	**1/2**	**0.49**		**0.00**	**-8.19**	
	3/2	1.10		1.09	-7.92	
	5/2	1.54		1.82	-7.46	
$CoN_3C_{10,porp}$	0	0.00		0.03	-6.97	
	**1**	**0.58**		**0.00**	**-6.81**	
	2	2.31		0.22	-6.45	
CoN_2C_5	1/2	1.94		0.15	-6.77	
	**3/2**	**1.83**		**0.00**	**-6.81**	
	5/2	2.17		1.44	-6.45	
O_2-CoN_4C_{12}	**1/2**	**-0.30**	**0.58 / 0.72**	**0.00**		**-0.80**
	3/2	0.16	0.57 / 0.66	0.24		-0.56
	5/2	1.28	0.78 / 0.85	0.29		-0.50
O_2-CoN_4C_{10}	**1/2**	**0.06**	**0.48 / 0.58**	**0.00**		**-0.97**
	3/2	0.69	0.74 / 0.78	0.50		-0.46
	5/2	1.22	0.83 / 0.87	1.51		-0.54
O_2-$CoN_3C_{10,porp}$	0	0.00	0.00 / 0.00	0.06		-1.18
	**1**	**0.05**	**0.20 / 0.33**	**0.00**		**-1.23**
	3	2.10	0.48 / 0.51	0.14		-1.09
O_2-CoN_2C_5	1/2	1.70	0.09 / 0.02	0.15		-1.11
	**3/2**	**1.83**	**0.21 / 0.18**	**0.00**		**-1.26**
	5/2	2.39	0.65 / 0.79	0.39		-0.87

The ground state structures are indicated in bold font.

Supplementary Table 5. Three possible cobalt site distributions leading to an average spin of 1.33 for a cobalt catalyst. These three cobalt moieties can individually match the XANES and EXAFS spectra of $Co_{0.5}$.

O_2-CoN_2C_5 Fraction / %	$CoN_3C_{10,porp}$ Fraction / %	CoN_4C_{12} Fraction / %	Average spin density of cobalt
Individual spin 1.83	Individual spin 0.58	Individual spin 0.88	-
60	40	0	1.330
47	0	53	1.326
53	16	31	1.335

The spin density of cobalt on each type of moiety in its ground-state (as reported in Supplementary Table 4) was considered to calculate the average spin.
